# Best Practices for Developing and Validating Scales for Health, Social, and Behavioral Research: A Primer

**DOI:** 10.3389/fpubh.2018.00149

**Published:** 2018-06-11

**Authors:** Godfred O. Boateng, Torsten B. Neilands, Edward A. Frongillo, Hugo R. Melgar-Quiñonez, Sera L. Young

**Affiliations:** ^1^Department of Anthropology and Global Health, Northwestern University, Evanston, IL, United States; ^2^Division of Prevention Science, Department of Medicine, University of California, San Francisco, San Francisco, CA, United States; ^3^Department of Health Promotion, Education and Behavior, Arnold School of Public Health, University of South Carolina, Columbia, SC, United States; ^4^Institute for Global Food Security, School of Human Nutrition, McGill University, Montreal, QC, Canada; ^5^Institute for Policy Research, Northwestern University, Evanston, IL, United States

**Keywords:** scale development, psychometric evaluation, content validity, item reduction, factor analysis, tests of dimensionality, tests of reliability, tests of validity

## Abstract

Scale development and validation are critical to much of the work in the health, social, and behavioral sciences. However, the constellation of techniques required for scale development and evaluation can be onerous, jargon-filled, unfamiliar, and resource-intensive. Further, it is often not a part of graduate training. Therefore, our goal was to concisely review the process of scale development in as straightforward a manner as possible, both to facilitate the development of new, valid, and reliable scales, and to help improve existing ones. To do this, we have created a primer for best practices for scale development in measuring complex phenomena. This is not a systematic review, but rather the amalgamation of technical literature and lessons learned from our experiences spent creating or adapting a number of scales over the past several decades. We identified three phases that span nine steps. In the first phase, items are generated and the validity of their content is assessed. In the second phase, the scale is constructed. Steps in scale construction include pre-testing the questions, administering the survey, reducing the number of items, and understanding how many factors the scale captures. In the third phase, scale evaluation, the number of dimensions is tested, reliability is tested, and validity is assessed. We have also added examples of best practices to each step. In sum, this primer will equip both scientists and practitioners to understand the ontology and methodology of scale development and validation, thereby facilitating the advancement of our understanding of a range of health, social, and behavioral outcomes.

## Introduction

Scales are a manifestation of latent constructs; they measure behaviors, attitudes, and hypothetical scenarios we expect to exist as a result of our theoretical understanding of the world, but cannot assess directly (1). Scales are typically used to capture a behavior, a feeling, or an action that cannot be captured in a single variable or item. The use of multiple items to measure an underlying latent construct can additionally account for, and isolate, item-specific measurement error, which

leads to more accurate research findings. Thousands of scales have been developed that can measure a range of social, psychological, and health behaviors and experiences.

As science advances and novel research questions are put forth, new scales become necessary. Scale development is not, however, an obvious or a straightforward endeavor. There are many steps to scale development, there is significant jargon within these techniques, the work can be costly and time consuming, and complex statistical analysis is often required. Further, many health and behavioral science degrees do not include training on scale development. Despite the availability of a large amount of technical literature on scale theory and development (1–7), there are a number of incomplete scales used to measure mental, physical, and behavioral attributes that are fundamental to our scientific inquiry (8, 9).

Therefore, our goal is to describe the process for scale development in as straightforward a manner as possible, both to facilitate the development of new, valid, and reliable scales, and to help improve existing ones. To do this, we have created a primer for best practices for scale development. We anticipate this primer will be broadly applicable across many disciplines, especially for health, social, and behavioral sciences. This is not a systematic review, but rather the amalgamation of technical literature and lessons learned from our experiences spent creating or adapting a number of scales related to multiple disciplines (10–23).

First, we provide an overview of each of the nine steps. Then, within each step, we define key concepts, describe the tasks required to achieve that step, share common pitfalls, and draw on examples in the health, social, and behavioral sciences to recommend best practices. We have tried to keep the material as straightforward as possible; references to the body of technical work have been the foundation of this primer.

## Scale development overview

**Figure 1:**
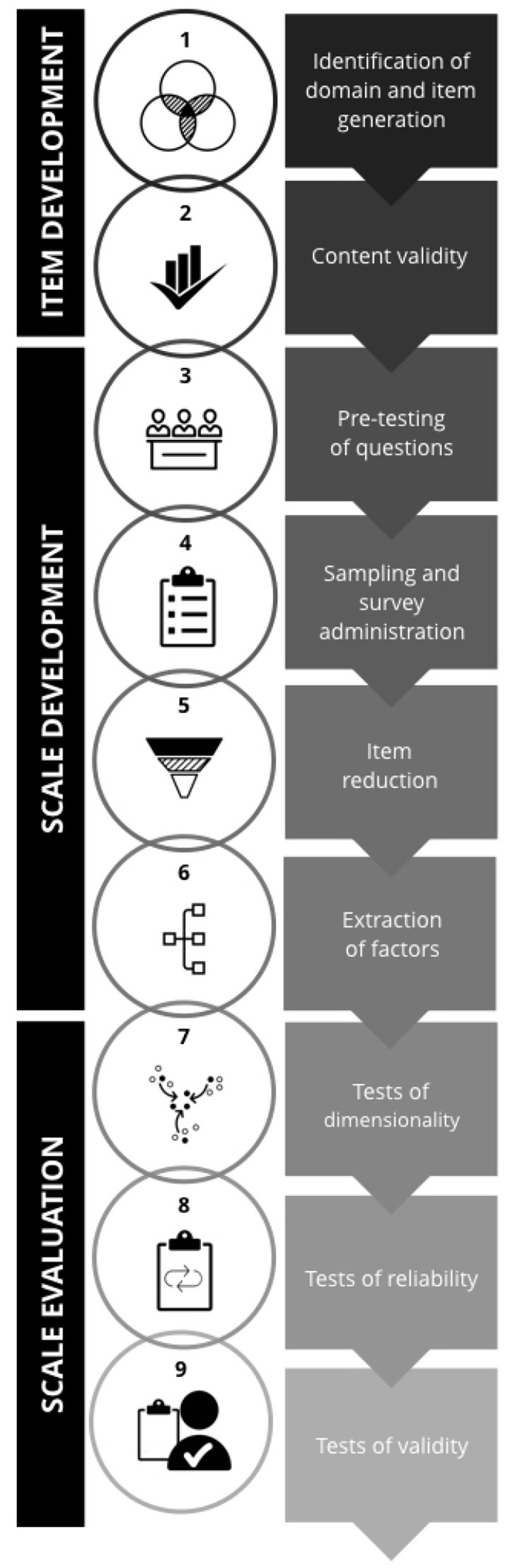
An overview of the three phases and nine steps of scale development and validation.

There are three phases to creating a rigorous scale—item development, scale development, and scale evaluation (24); these can be further broken down into nine steps (Figure 1).

Item development, i.e., coming up with the initial set of questions for an eventual scale, is composed of: (1) identification of the domain(s) and item generation, and (2) consideration of content validity. The second phase, scale development, i.e., turning individual items into a harmonious and measuring construct, consists of (3) pre-testing questions, (4) sampling and survey administration, (5) item reduction, and (6) extraction of latent factors. The last phase, scale evaluation, requires: (7) tests of dimensionality, (8) tests of reliability, and (9) tests of validity.

**Table 1:** The three phases and nine steps of scale development and validation.

**Activity**	**Purpose**	**How to explore or estimate?**	**References**
**PHASE 1: ITEM DEVELOPMENT**			
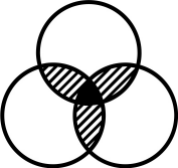	**Step 1: Identification of Domain and Item Generation: Selecting Which Items to Ask**		
Domain identification	To specify the boundaries of the domain and facilitate item generation	1.1 Specify the purpose of the domain 1.2 Confirm that there are no existing instruments 1.3 Describe the domain and provide preliminary conceptual definition 1.4 Specify the dimensions of the domain if they exist *a priori* 1.5 Define each dimension	(1–4), (25)
Item generation	To identify appropriate questions that fit the identified domain	1.6 Deductive methods: literature review and assessment of existing scales 1.7 Inductive methods: exploratory research methodologies including focus group discussions and interviews	(2–5), (24–41)
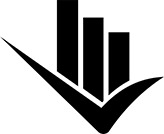	**Step 2: Content Validity: Assessing if the Items Adequately Measure the Domain of Interest**		
Evaluation by experts	To evaluate each of the items constituting the domain for content relevance, representativeness, and technical quality	2.1 Quantify assessments of 5-7 expert judges using formalized scaling and statistical procedures including content validity ratio, content validity index, or Cohen's coefficient alpha 2.2 Conduct Delphi method with expert judges	(1–5), (24, 42–48)
Evaluation by target population	To evaluate each item constituting the domain for representativeness of actual experience from target population	2.3 Conduct cognitive interviews with end users of scale items to evaluate face validity	(20, 25)
**PHASE 2: SCALE DEVELOPMENT**			
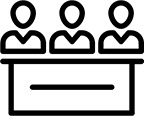	**Step 3: Pre-testing Questions: Ensuring the Questions and Answers Are Meaningful**		
Cognitive interviews	To assess the extent to which questions reflect the domain of interest and that answers produce valid measurements	3.1 Administer draft questions to 5–15 interviewees in 2–3 rounds while allowing respondents to verbalize the mental process entailed in providing answers	(49–54)
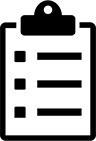	**Step 4: Survey Administration and Sample Size: Gathering Enough Data from the Right People**		
Survey administration	To collect data with minimum measurement errors	4.1 Administer potential scale items on a sample that reflects range of target population using paper or device	(55–58)
Establishing the sample size	To ensure the availability of sufficient data for scale development	4.2 Recommended sample size is 10 respondents per survey item and/or 200-300 observations	(29, 59–65)
Determining the type of data to use	To ensure the availability of data for scale development and validation	4.3 Use cross-sectional data for exploratory factor analysis 4.4 Use data from a second time point, at least 3 months later in a longitudinal dataset, or an independent sample for test of dimensionality (Step 7)	–
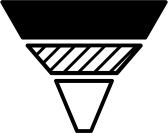	**Step 5: Item Reduction: Ensuring Your Scale Is Parsimonious**		
Item difficulty index	To determine the proportion of correct answers given per item (CTT) To determine the probability of a particular examinee correctly answering a given item (IRT)	5.1 Proportion can be calculated for CTT and item difficulty parameter estimated for IRT using statistical packages	(1, 2, 66–68)
Item discrimination test	To determine the degree to which an item or set of test questions are measuring a unitary attribute (CTT) To determine how steeply the probability of correct response changes as ability increases (IRT)	5.2 Estimate biserial correlations or item discrimination parameter using statistical packages	(69–75)
Inter-item and item-total correlations	To determine the correlations between scale items, as well as the correlations between each item and sum score of scale items	5.3 Estimate inter-item/item communalities, item-total, and adjusted item-total correlations using statistical packages	(1, 2, 68, 76)
Distractor efficiency analysis	To determine the distribution of incorrect options and how they contribute to the quality of items	5.4 Estimate distractor analysis using statistical packages	(77–80)
Deleting or imputing missing cases	To ensure the availability of complete cases for scale development	5.5 Delete items with many cases that are permanently missing, or use multiple imputation or full information maximum likelihood for imputation of data	(81–84)
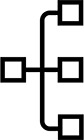	**Step 6: Extraction of Factors: Exploring the Number of Latent Constructs that Fit Your Observed Data**		
Factor analysis	To determine the optimal number of factors or domains that fit a set of items	6.1 Use scree plots, exploratory factor analysis, parallel analysis, minimum average partial procedure, and/or the Hull method	(2–4), (85–90)
**PHASE 3: SCALE EVALUTION**			
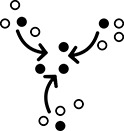	**Step 7: Tests of Dimensionality: Testing if Latent Constructs Are as Hypothesized**		
Test dimensionality	To address queries on the latent structure of scale items and their underlying relationships. i.e., to validate whether the previous hypothetical structure fits the items	7.1 Estimate independent cluster model—confirmatory factor analysis, cf. Table 2 7.2 Estimate bifactor models to eliminate ambiguity about the type of dimensionality—unidimensionality, bidimensionality, or multi-dimensionality 7.3 Estimate measurement invariance to determine whether hypothesized factor and dimension is congruent across groups or multiple samples	(91–114)
Score scale items	To create scale scores for substantive analysis including reliability and validity of scale	7.4. calculate scale scores using an unweighted approach, which includes summing standardized item scores and raw item scores, or computing the mean for raw item scores 7.5. Calculate scale scores by using a weighted approach, which includes creating factor scores via confirmatory factor analysis or structural equation models	(115)
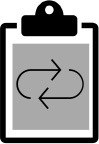	**Step 8: Tests of Reliability: Establishing if Responses Are Consistent When Repeated**		
Calculate reliability statistics	To assess the internal consistency of the scale. i.e., the degree to which the set of items in the scale co-vary, relative to their sum score	8.1 Estimate using Cronbach's alpha 8.2. Other tests such as Raykov's rho, ordinal alpha, and Revelle's beta can be used to assess scale reliability	(116–123)
Test–retest reliability	To assess the degree to which the participant's performance is repeatable; i.e., how consistent their scores are across time	8.3 Estimate the strength of the relationship between scale items over two or three time points; variety of measures possible	(1, 2, 124, 125)
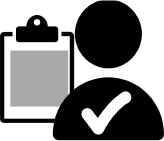	**Step 9: Tests of Validity: Ensuring You Measure the Latent Dimension You Intended**		
**Criterion validity**			
Predictive validity	To determine if scores predict future outcomes	9.1 Use bivariate and multivariable regression; stronger and significant associations or causal effects suggest greater predictive validity	(1, 2, 31)
Concurrent validity	To determine the extent to which scale scores have a stronger relationship with criterion measurements made near the time of administration	9.2 Estimate the association between scale scores and “gold standard” of scale measurement; stronger significant association in Pearson product-moment correlation suggests support for concurrent validity	(2)
**Construct validity**			
Convergent validity	To examine if the same concept measured in different ways yields similar results	9.3 Estimate the relationship between scale scores and similar constructs using multi-trait multi-method matrix, latent variable modeling, or Pearson product-moment coefficient; higher/stronger correlation coefficients suggest support for convergent validity	(2, 37, 126)
Discriminant validity	To examine if the concept measured is different from some other concept	9.4 Estimate the relationship between scale scores and distinct constructs using multi-trait multi-method matrix, latent variable modeling, or Pearson product-moment coefficient; lower/weaker correlation coefficients suggest support for discriminant validity	(2, 37, 126)
Differentiation by “known groups”	To examine if the concept measured behaves as expected in relation to “known groups”	9.5 Select known binary variables based on theoretical and empirical knowledge and determine the distribution of the scale scores over the known groups; use *t*-tests if binary, ANOVA if multiple groups	(2, 126)
Correlation analysis	To determine the relationship between existing measures or variables and newly developed scale scores	9.6 Correlate scale scores and existing measures or, preferably, use linear regression, intraclass correlation coefficient, and analysis of standard deviations of the differences between scores	(2, 127, 128)

## Phase 1: item development

### Step 1: identification of the domain(s) and item generation

#### Domain identification

The first step is to articulate the domain(s) that you are endeavoring to measure. A domain or construct refers to the concept, attribute, or unobserved behavior that is the target of the study (25). Therefore, the domain being examined should be decided upon and defined before any item activity (2). A well-defined domain will provide a working knowledge of the phenomenon under study, specify the boundaries of the domain, and ease the process of item generation and content validation.

McCoach et al. outline a number of steps in scale development; we find the first five to be suitable for the identification of domain (4). These are all based on thorough literature review and include (a) specifying the purpose of the domain or construct you seek to develop, and (b), confirming that there are no existing instruments that will adequately serve the same purpose. Where there is a similar instrument in existence, you need to justify why the development of a new instrument is appropriate and how it will differ from existing instruments. Then, (c) describe the domain and provide a preliminary conceptual definition and (d) specify, if any, the dimensions of the domain. Alternatively, you can let the number of dimensions forming the domain to be determined through statistical computation (cf. Steps 5, 6, and 7). Domains are determined *a priori* if there is an established framework or theory guiding the study, but *a posteriori* if none exist. Finally, if domains are identified *a priori*, (e) the final conceptual definition for each domain should be specified.

#### Item generation

Once the domain is delineated, the item pool can then be identified. This process is also called “question development” (26) or “item generation” (24). There are two ways to identify appropriate questions: deductive and inductive methods (24).

The deductive method, also known as “logical partitioning” or “classification from above” (27) is based on the description of the relevant domain and the identification of items. This can be done through literature review and assessment of existing scales and indicators of that domain (2, 24). The inductive method, also known as “grouping” or “classification from below” (24, 27) involves the generation of items from the responses of individuals (24). Qualitative data obtained through direct observations and exploratory research methodologies, such as focus groups and individual interviews, can be used to inductively identify domain items (5).

It is considered best practice to combine both deductive and inductive methods to both define the domain and identify the questions to assess it. While the literature review provides the theoretical basis for defining the domain, the use of qualitative techniques moves the domain from an abstract point to the identification of its manifest forms. A scale or construct defined by theoretical underpinnings is better placed to make specific pragmatic decisions about the domain (28), as the construct will be based on accumulated knowledge of existing items.

It is recommended that the items identified using deductive and inductive approaches should be broader and more comprehensive than one's own theoretical view of the target (28, 29). Further, content should be included that ultimately will be shown to be tangential or unrelated to the core construct. In other words, one should not hesitate to have items on the scale that do not perfectly fit the domain identified, as successive evaluation will eliminate undesirable items from the initial pool. Kline and Schinka et al. note that the initial pool of items developed should be at minimum twice as long as the desired final scale (26, 30). Others have recommended the initial pool to be five times as large as the final version, to provide the requisite margin to select an optimum combination of items (30). We agree with Kline and Schinka et al. (26, 30) that the number of items should be at least twice as long as the desired scale.

Further, in the development of items, the *form* of the items, the *wording of the items*, and the types of *responses* that the question is designed to induce should be taken into account. It also means questions should capture the lived experiences of the phenomenon by target population (30). Further, items should be worded simply and unambiguously. Items should not be offensive or potentially biased in terms of social identity, i.e., gender, religion, ethnicity, race, economic status, or sexual orientation (30).

Fowler identified five essential characteristics of items required to ensure the quality of construct measurement (31). These include (a) the need for items to be consistently understood; (b) the need for items to be consistently administered or communicated to respondents; (c) the consistent communication of what constitutes an adequate answer; (d) the need for all respondents to have access to the information needed to answer the question accurately; and (e) the willingness for respondents to provide the correct answers required by the question at all times.

These essentials are sometimes very difficult to achieve. Krosnick (32) suggests that respondents can be less thoughtful about the meaning of a question, search their memories less comprehensively, integrate retrieved information less carefully, or even select a less precise response choice. All this means that they are merely satisficing, i.e., providing merely satisfactory answers, rather than the most accurate ones. In order to combat this behavior, questions should be kept simple, straightforward, and should follow the conventions of normal conversation.

With regards to the type of responses to these questions, we recommend that questions with dichotomous response categories (e.g., true/false) should have no ambiguity. When a Likert-type response scale is used, the points on the scale should reflect the entire measurement continuum. Responses should be presented in an ordinal manner, i.e., in an ascending order without any overlap, and each point on the response scale should be meaningful and interpreted the same way by each participant to ensure data quality (33).

In terms of the number of points on the response scale, Krosnick and Presser (33) showed that responses with just two to three points have lower reliability than Likert-type response scales with five to seven points. However, the gain levels off after seven points. Therefore, response scales with five points are recommended for unipolar items, i.e., those reflecting relative degrees of a single item response quality, e.g., not at all satisfied to very satisfied. Seven response items are recommended for bipolar items, i.e., those reflecting relative degrees of two qualities of an item response scale, e.g., completely dissatisfied to completely satisfied. As an analytic aside, items with scale points fewer than five categories are best estimated using robust categorical methods. However, items with five to seven categories without strong floor or ceiling effects can be treated as continuous items in confirmatory factor analysis and structural equation modeling using maximum likelihood estimations (34).

One pitfall in the identification of domain and item generation is the improper conceptualization and definition of the domain(s). This can result in scales that may either be deficient because the definition of the domain is ambiguous or has been inadequately defined (35). It can also result in contamination, i.e., the definition of the domain overlaps with other existing constructs in the same field (35).

Caution should also be taken to avoid construct underrepresentation, which is when a scale does not capture important aspects of a construct because its focus is too narrow (35, 36). Further, construct-irrelevant variance, which is the degree to which test scores are influenced by processes that have little to do with the intended construct and seem to be widely inclusive of non-related items (36, 37), should be avoided. Both construct underrepresentation and irrelevant variance can lead to the invalidation of the scale (36).

An example of best practice using the deductive approach to item generation is found in the work of Dennis on breastfeeding self-efficacy (38–40). Dennis' breastfeeding self-efficacy scale items were first informed by Bandura's theory on self-efficacy, followed by content analysis of literature review, and empirical studies on breastfeeding-related confidence.

A valuable example for a rigorous inductive approach is found in the work of Frongillo and Nanama on the development and validation of an experience-based measure of household food insecurity in northern Burkina Faso (41). In order to generate items for the measure, they undertook in-depth interviews with 10 household heads and 26 women using interview guides. The data from these interviews were thematically analyzed, with the results informing the identification of items to be added or deleted from the initial questionnaire. Also, the interviews led to the development and revision of answer choices.

### Step 2: content validity

Content validity, also known as “theoretical analysis” (5), refers to the “adequacy with which a measure assesses the domain of interest” (24). The need for content adequacy is vital if the items are to measure what they are presumed to measure (1). Additionally, content validity specifies content relevance and content representations, i.e., that the items capture the relevant experience of the target population being examined (129).

Content validity entails the process of ensuring that only the phenomenon spelled out in the conceptual definition, but not other aspects that “might be related but are outside the investigator's intent for that particular [construct] are added” (1). Guion has proposed five conditions that must be satisfied in order for one to claim any form of content validity. We find these conditions to be broadly applicable to scale development in any discipline. These include that (a) the behavioral content has a generally accepted meaning or definition; (b) the domain is unambiguously defined; (c) the content domain is relevant to the purposes of measurement; (d) qualified judges agree that the domain has been adequately sampled based on consensus; and (e) the response content must be reliably observed and evaluated (42). Therefore, content validity requires evidence of content relevance, representativeness, and technical quality.

Content validity is mainly assessed through evaluation by expert and target population judges.

#### Evaluation by experts

Expert judges are highly knowledgeable about the domain of interest and/or scale development; target population judges are potential users of the scale (1, 5). Expert judges seem to be used more often than target-population judges in scale development work to date. Ideally, one should combine expert and target population judgment. When resources are constrained, however, we recommend *at least* the use of expert judges.

Expert judges evaluate each of the items to determine whether they represent the domain of interest. These expert judges should be independent of those who developed the item pool. Expert judgment can be done systematically to avoid bias in the assessment of items. Multiple judges have been used (typically ranging from 5 to 7) (25). Their assessments have been quantified using formalized scaling and statistical procedures such as the content validity ratio for quantifying consensus (43), content validity index for measuring proportional agreement (44), or Cohen's coefficient kappa (*k*) for measuring inter-rater or expert agreement (45). Among the three procedures, we recommend Cohen's coefficient kappa, which has been found to be most efficient (46). Additionally, an increase in the number of experts has been found to increase the robustness of the ratings (25, 44).

Another way by which content validity can be assessed through expert judges is by using the Delphi method to come to a consensus on which questions are a reflection of the construct you want to measure. The Delphi method is a technique “for structuring group communication process so that the process is effective in allowing a group of individuals, as a whole, to deal with a complex problem” (47).

A good example of evaluation of content validity using expert judges is seen in the work of Augustine et al. on adolescent knowledge of micronutrients (48). After identifying a list of items to be validated, the authors consulted experts in the field of nutrition, psychology, medicine, and basic sciences. The items were then subjected to content analysis using expert judges. Two independent reviews were carried out by a panel of five experts to select the questions that were appropriate, accurate, and interpretable. Items were either accepted, rejected, or modified based on majority opinion (48).

#### Evaluation by target population

Target population judges are experts at evaluating face validity, which is a component of content validity (25). Face validity is the “degree that respondents or end users [or lay persons] judge that the items of an assessment instrument are appropriate to the targeted construct and assessment objectives” (25). These end-users are able to tell whether the construct is a good measure of the domain through cognitive interviews, which we discuss in Step 3.

An example of the concurrent use of expert and target population judges comes from Boateng et al.'s work to develop a household-level water insecurity scale appropriate for use in western Kenya (20). We used the Delphi method to obtain three rounds of feedback from international experts including those in hydrology, geography, WASH and water-related programs, policy implementation, and food insecurity. Each of the three rounds was interspersed with focus group discussions with our target population, i.e., people living in western Kenya. In each round, the questionnaires progressively became more closed ended, until consensus was attained on the definition of the domain we were studying and possible items we could use.

## Phase 2: scale development

### Step 3: pre-testing questions

Pre-testing helps to ensure that items are meaningful to the target population before the survey is actually administered, i.e., it minimizes misunderstanding and subsequent measurement error. Because pre-testing eliminates poorly worded items and facilitates revision of phrasing to be maximally understood, it also serves to reduce the cognitive burden on research participants. Finally, pre-testing represents an additional way in which members of the target population can participate in the research process by contributing their insights to the development of the survey.

Pre-testing has two components: the first is the examination of the extent to which the questions reflect the domain being studied. The second is the examination of the extent to which answers to the questions asked produce valid measurements (31).

#### Cognitive interviews

To evaluate whether the questions reflect the domain of study and meet the requisite standards, techniques including cognitive interviews, focus group discussion, and field pre-testing under realistic conditions can be used. We describe the most recommended, which is cognitive interviews.

Cognitive interviewing entails the administration of draft survey questions to target populations and then asking the respondents to verbalize the mental process entailed in providing such answers (49). Generally, cognitive interviews allow for questions to be modified, clarified, or augmented to fit the objectives of the study. This approach helps to determine whether the question is generating the information that the author intends by helping to ensure that respondents understand questions as developers intended and that respondents are able to answer in a manner that reflects their experience (49, 50). This can be done on a sample outside of the study population or on a subset of study participants, but it must be explored before the questionnaire is finalized (51, 52).

The sample used for cognitive interviewing should capture the range of demographics you anticipate surveying (49). A range of 5–15 interviews in two to three rounds, or until saturation, or relatively few new insights emerge is considered ideal for pre-testing (49, 51, 52).

In sum, cognitive interviews get to the heart of both assessing the appropriateness of the question to the target population *and* the strength of the responses (49). The advantages of using cognitive interviewing include: (a) it ensures questions are producing the intended data, (b) questions that are confusing to participants are identified and improved for clarity, (c) problematic questions or questions that are difficult to answer are identified, (d) it ensures response options are appropriate and adequate, (e) it reveals the thought process of participants on domain items, and (f) it can indicate problematic question order (52, 53). Outcomes of cognitive interviews should always be reported, along with solutions used to remedy the situation.

An example of best practice in pre-testing is seen in the work of Morris et al. (54). They developed and validated a novel scale for measuring interpersonal factors underlying injection drug use behaviors among injecting partners. After item development and expert judgment, they conducted cognitive interviews with seven respondents with similar characteristics to the target population to refine and assess item interpretation and to finalize item structure. Eight items were dropped after cognitive interviews for lack of clarity or importance. They also made modifications to grammar, word choice, and answer options based on the feedback from cognitive interviews.

### Step 4: survey administration and sample size

#### Survey administration

Collecting data with minimum measurement errors from an adequate sample size is imperative. These data can be collected using paper and pen/pencil interviewing (PAPI) or Computer Assisted Personal Interviewing (CAPI) on devices like laptops, tablets, or phones. A number of software programs exist for building forms on devices. These include Computer Assisted Survey Information Collection (CASIC) Builder™ (West Portal Software Corporation, San Francisco, CA); Qualtrics Research Core™ (www.qualtrics.com); Open Data Kit (ODK, https://opendatakit.org/); Research Electronic Data Capture (REDCap) (55); SurveyCTO (Dobility, Inc. https://www.surveycto.com); and Questionnaire Development System™ (QDS, www.novaresearch.com), which allows the participant to report sensitive audio data.

Each approach has advantages and drawbacks. Using technology can reduce the errors associated with data entry, allow the collection of data from large samples with minimal cost, increase response rate, reduce enumerator errors, permit instant feedback, and increase monitoring of data collection and ability to get more confidential data (56–58, 130). A subset of technology-based programs offers the option of attaching audio files to the survey questions so that questions may be recorded and read out loud to participants with low literacy via audio computer self-assisted interviewing (A-CASI) (131). Self-interviewing, whether via A-CASI or via computer-assisted personal interviewing, in which participants read and respond to questions on a computer without interviewer involvement, may increase reports of sensitive or stigmatized behaviors such as sexual behaviors and substance use, compared to when being asked by another human.

On the other hand, paper forms may avert the crisis of losing data if the software crashes, the devices are lost or stolen prior to being backed up, and may be more suitable in areas that have irregular electricity and/or internet. However, as sample sizes increase, the use of PAPI becomes more expensive, time and labor intensive, and the data are exposed in several ways to human error (57, 58). Based on the merits of CAPI over PAPI, we recommend researchers use CAPI in data collection for surveys when feasible.

#### Establishing the sample size

The sample size to use for the development of a latent construct has often been contentious. It is recommended that potential scale items be tested on a heterogeneous sample, i.e., a sample that both reflects and captures the range of the target population (29). For example, when the scale is used in a clinical setting, Clark and Watson recommend using patient samples early on instead of a sample from the general population (29).

The necessary sample size is dependent on several aspects of any given study, including the level of variation between the variables, and the level of over-determination (i.e., the ratio of variables to number of factors) of the factors (59). The rule of thumb has been at least 10 participants for each scale item, i.e., an ideal ratio of respondents to items is 10:1 (60). However, others have suggested sample sizes that are independent of the number of survey items. Clark and Watson (29) propose using 300 respondents after initial pre-testing. Others have recommended a range of 200–300 as appropriate for factor analysis (61, 62). Based on their simulation study using different sample sizes, Guadagnoli and Velicer (61) suggested that a minimum of 300–450 is required to observe an acceptable comparability of patterns, and that replication is required if the sample size is < 300. Comrey and Lee suggest a graded scale of sample sizes for scale development: 100 = poor, 200 = fair, 300 = good, 500 = very good, ≥1,000 = excellent (63). Additionally, item reduction procedures (described, below in Step 5), such as parallel analysis which requires bootstrapping (estimating statistical parameters from sample by means of resampling with replacement) (64), may require larger data sets.

In sum, there is no single item-ratio that works for all survey development scenarios. A larger sample size or respondent: item ratio is always better, since a larger sample size implies lower measurement errors and more, stable factor loadings, replicable factors, and generalizable results to the true population structure (59, 65). A smaller sample size or respondent: item ratio may mean more unstable loadings and factors, random, non-replicable factors, and non-generalizable results (59, 65). Sample size is, however, always constrained by resources available, and more often than not, scale development can be difficult to fund.

#### Determining the type of data to use

The development of a scale minimally requires data from a single point in time. To fully test for the reliability of the scale (cf. Steps 8, 9), however, either an independent dataset or a subsequent time point is necessary. Data from longitudinal studies can be used for initial scale development (e.g., from baseline), to conduct confirmatory factor analysis (using follow-up data, cf. Step 7), and to assess test–retest reliability (using baseline and follow-up data). The problem with using longitudinal data to test hypothesized latent structures is common error variance, since the same, potentially idiosyncratic, participants will be involved. To give the most credence to the reliability of scale, the ideal procedure is to develop the scale on sample A, whether cross-sectional or longitudinal, and then test it on an independent sample B.

The work of Chesney et al. on the Coping Self-Efficacy scale provides an example of this best practice in the use of independent samples (132). This study sought to investigate the psychometric characteristics of the Coping Self-Efficacy (CSE) scale, and their samples came from two independent randomized clinical trials. As such, two independent samples with four different time points each (0, 3, 6, and 12 months) were used. The authors administered the 26-item scale to the sample from the first clinical trial and examined the covariance that existed between all the scale items (exploratory factor analysis) giving the hypothesized factor structure across time in that one trial. The obtained factor structure was then fitted to baseline data from the second randomized clinical trial to test the hypothesized factor structure generated in the first sample (132).

### Step 5: item reduction analysis

In scale development, item reduction analysis is conducted to ensure that only parsimonious, functional, and internally consistent items are ultimately included (133). Therefore, the goal of this phase is to identify items that are not or are the least-related to the domain under study for deletion or modification.

Two theories, Classical Test Theory (CTT) and the Item Response Theory (IRT), underpin scale development (134). CTT is considered the traditional test theory and IRT the modern test theory; both function to produce latent constructs. Each theory may be used singly or in conjunction to complement the other's strengths (15, 135). Whether the researcher is using CTT or IRT, the primary goal is to obtain functional items (i.e., items that are correlated with each other, discriminate between individual cases, underscore a single or multidimensional domain, and contribute significantly to the construct).

CTT allows the prediction of outcomes of constructs and the difficulty of items (136). CTT models assume that items forming constructs in their observed, manifest forms consist of a true score on the domain of interest and a random error (which is the differences between the true score and a set of observed scores by an individual) (137). IRT seeks to model the way in which constructs manifest themselves in terms of observable item response (138). Comparatively, the IRT approach to scale development has the advantage of allowing the researcher to determine the effect of adding or deleting a given item or set of items by examining the item information and standard error functions for the item pool (138).

Several techniques exist within the two theories to reduce the item pool, depending on which test theory is driving the scale. The five major techniques used are: item difficulty and item discrimination indices, which are primarily for binary responses; inter-item and item-total correlations, which are mostly used for categorical items; and distractor efficiency analysis for items with multiple choice response options (1, 2).

#### Item difficulty index

The item difficulty index is both a CTT and an IRT parameter that can be traced largely to educational and psychological testing to assess the relative difficulties and discrimination abilities of test items (66). Subsequently, this approach has been applied to more attitudinal-type scales designed to measure latent constructs.

Under the CTT framework, the item difficulty index, also called item easiness, is the proportion of correct answers on a given item, e.g., the proportion of correct answers on a math test (1, 2). It ranges between 0.0 and 1.0. A high difficulty score means a greater proportion of the sample answered the question correctly. A lower difficulty score means a smaller proportion of the sample understood the question and answered correctly. This may be due to the item being coded wrongly, ambiguity with the item, confusing language, or ambiguity with response options. A lower difficulty score suggests a need to modify the items or delete them from the pool of items.

Under the IRT framework, the item difficulty parameter is the probability of a particular examinee correctly answering any given item (67). This has the advantage of allowing the researcher to identify the different levels of individual performance on specific questions, as well as develop particular questions to specific subgroups or populations (67). Item difficulty is estimated directly using logistic models instead of proportions.

Researchers must determine whether they need items with low, medium, or high difficulty. For instance, researchers interested in general purpose scales will focus on items with medium difficulty (68), i.e., the proportion with item assertions ranging from 0.4 to 0.6 (2, 68). The item difficulty index can be calculated using existing commands in M*plus*, R, SAS, SPSS, or Stata.

#### Item discrimination index

The item discrimination index (also called item-effectiveness test), is the degree to which an item correctly differentiates between respondents or examinees on a construct of interest (69), and can be assessed under both CTT and IRT frameworks. It is a measure of the difference in performance between groups on a construct. The upper group represents participants with high scores and the lower group those with poor or low scores. The item discrimination index is “calculated by subtracting the proportion of examinees in the lower group (lower %) from the proportion of examinees in the upper group (upper %) who got the item correct or endorsed the item in the expected manner” (69). It differentiates between the number of students in an upper group who get an item correct and the number of students in a lower group who get the item correct (70). The use of an item discrimination index enables the identification of positively discriminating items (i.e., items that differentiate rightly between those who are knowledgeable about a subject and those who are not), negatively discriminating items (i.e., items which are poorly designed such that the more knowledgeable get them wrong and the less knowledgeable get them right), and non-discriminating item (i.e., items that fail to differentiate between participants who are knowledgeable about a subject and those who are not) (70).

The item discrimination index has been found to improve test items in at least three ways. First, non-discriminating items, which fail to discriminate between respondents because they may be too easy, too hard, or ambiguous, should be removed (71). Second, items which negatively discriminate, e.g., items which fail to differentiate rightly between medically diagnosed depressed and non-depressed respondents on a happiness scale, should be reexamined and modified (70, 71). Third, items that positively discriminate should be retained, e.g., items that are correctly affirmed by a greater proportion of respondents who are medically free of depression, with very low affirmation by respondents diagnosed to be medically depressed (71). In some cases, it has been recommended that such positively discriminating items be considered for revision (70) as the differences could be due to the level of difficulty of the item.

An item discrimination index can be calculated through correlational analysis between the performance on an item and an overall criterion (69) using either the point biserial correlation coefficient or the phi coefficient (72).

Item discrimination under the IRT framework is a slope parameter that determines how steeply the probability of a correct response changes as the proficiency or trait increases (73). This allows differentiation between individuals with similar abilities and can also be estimated using a logistic model. Under certain conditions, the biserial correlation coefficient under the CTT framework has proven to be identical to the IRT item discrimination parameter (67, 74, 75); thus, as the trait increases so does the probability of endorsing an item. These parameters can be computed using existing commands in M*plus*, R, SAS, SPSS, or Stata. In both CTT and IRT, higher values are indicators of greater discrimination (73).

#### Inter-item and item-total correlations

A third technique to support the deletion or modification of items is the estimation of inter-item and item-total correlations, which falls under CTT. These correlations often displayed in the form of a matrix are used to examine relationships that exist between individual items in a pool.

Inter-item correlations (also known as polychoric correlations for categorical variables and tetrachoric correlations for binary items) examines the extent to which scores on one item are related to scores on all other items in a scale (2, 68, 76). Also, it examines the extent to which items on a scale are assessing the same content (76). Items with very low correlations (< 0.30) are less desirable and could be a cue for potential deletion from the tentative scale.

Item-total correlations (also known as polyserial correlations for categorical variables and biserial correlations for binary items) aim at examining the relationship between each item vs. the total score of scale items. However, the adjusted item-total correlation, which examines the correlation between the item and the sum score of the rest of the items excluding itself is preferred (1, 2). Items with very low adjusted item-total correlations (< 0.30) are less desirable and could be a cue for potential deletion from the tentative scale. Inter-item and item total correlations can be calculated using M*plus*, R, SAS, SPSS, or Stata.

#### Distractor efficiency analysis

The distractor efficiency analysis shows the distribution of incorrect options and how they contribute to the quality of a multiple-choice item (77). The incorrect options, also known as distractors, are intentionally added in the response options to attract students who do not know the correct answer in a test question (78). To calculate this, respondents will be grouped into three groups—high, middle, and lower tertiles based on their total scores on a set of items. Items will be regarded as appropriate if 100% of those in the high group choose the correct response options, about 50% of those in the middle choose the correct option, and few or none in the lower group choose the correct option (78). This type of analysis is rarely used in the health sciences, as most multiple-choice items are on a Likert-type response scale and do not test respondent correct knowledge, but their experience or perception. However, distractor analysis can help to determine whether items are well-constructed, meaningful, and functional when researchers add response options to questions that do not fit a particular experience. It is expected that participants who are determined as having poor knowledge or experience on the construct will choose the distractors, while those with the right knowledge and experience will choose the correct response options (77, 79). Where those with the right knowledge and experience are not able to differentiate between distractors and the right response, the question may have to be modified. Non-functional distractors identified need to be removed and replaced with efficient distractors (80).

#### Missing cases

In addition to these techniques, some researchers opt to delete items with large numbers of cases that are missing, when other missing data-handling techniques cannot be used (81). For cases where modern missing data handling can be used, however, several techniques exist to solve the problem of missing cases. Two of the approaches have proven to be very useful for scale development: full information maximum likelihood (FIML) (82) and multiple imputation (83). Both methods can be applied using existing commands in statistical packages such as M*plus*, R, SAS, and Stata. When using multiple imputation to recover missing data in the context of survey research, the researcher can impute individual items prior to computing scale scores or impute the scale scores from other scale scores (84). However, item-level imputation has been shown to produce more efficient estimates over scale-level imputation. Thus, imputing individual items before scale development is a preferred approach to imputing newly developed scales for missing cases (84).

### Step 6: extraction of factors

Factor extraction is the phase in which the optimal number of factors, sometimes called domains, that fit a set of items are determined. This is done using factor analysis. Factor analysis is a regression model in which observed standardized variables are regressed on unobserved (i.e., latent) factors. Because the variables and factors are standardized, the bivariate regression coefficients are also correlations, representing the loading of each observed variable on each factor. Thus, factor analysis is used to understand the latent (internal) structure of a set of items, and the extent to which the relationships between the items are internally consistent (4). This is done by extracting latent factors which represent the shared variance in responses among the multiple items (4). The emphasis is on the number of factors, the salience of factor loading estimates, and the relative magnitude of residual variances (2).

A number of analytical processes have been used to determine the number of factors to retain from a list of items, and it is beyond the scope of this paper to describe all of them. For scale development, commonly available methods to determine the number of factors to retain include a scree plot (85), the variance explained by the factor model, and the pattern of factor loadings (2). Where feasible, researchers could also assess the optimal number of factors to be drawn from the list of items using either parallel analysis (86), minimum average partial procedure (87), or the Hull method (88, 89).

The extraction of factors can also be used to reduce items. With factor analysis, items with factor loadings or slope coefficients that are below 0.30 are considered inadequate as they contribute <10% variation of the latent construct measured. Hence, it is often recommended to retain items that have factor loadings of 0.40 and above (2, 60). Also, items with cross-loadings or that appear not to load uniquely on individual factors can be deleted. For single-factor models in which Rasch IRT modeling is used, items are selected as having a good fit based on mean-square residual summary statistics (infit and outfit) >0.4 and <1.6 (90).

A number of scales developed stop at this phase and jump to tests of reliability, but the factors extracted at this point only provide a *hypothetical* structure of the scale. The dimensionality of these factors need to be tested (cf. Step 7) before moving on to reliability (cf. Step 8) and validity (cf. Step 9) assessment.

## Phase 3: scale evaluation

### Step 7: tests of dimensionality

The test of dimensionality is a test in which the hypothesized factors or factor structure extracted from a previous model is tested at a different time point in a longitudinal study or, ideally, on a new sample (91). Tests of dimensionality determine whether the measurement of items, their factors, and function are the same across two independent samples or within the same sample at different time points. Such tests can be conducted using independent cluster model (ICM)-confirmatory factor analysis, bifactor modeling, or measurement invariance.

#### Confirmatory factor analysis

Confirmatory factor analysis is a form of psychometric assessment that allows for the systematic comparison of an alternative *a priori* factor structure based on systematic fit assessment procedures and estimates the relationship between latent constructs, which have been corrected for measurement errors (92). Morin et al. (92) note that it relies on a highly restrictive ICM, in which cross-loadings between items and non-target factors are assumed to be exactly zero. The systematic fit assessment procedures are determined by meaningful satisfactory thresholds; Table 2 contains the most common techniques for testing dimensionality. These techniques include the chi-square test of exact fit, Root Mean Square Error of Approximation (RMSEA ≤ 0.06), Tucker Lewis Index (TLI ≥ 0.95), Comparative Fit Index (CFI ≥ 0.95), Standardized Root Mean Square Residual (SRMR ≤ 0.08), and Weighted Root Mean Square Residual (WRMR ≤ 1.0) (90, 92–101).

**Table 2:** Description of model fit indices and thresholds for evaluating scales developed for health, social, and behavioral research.

**Model fit indices**	**Description**	**Recommended threshold to use**	**References**
Chi-square test	The chi-square value is a test statistic of the goodness of fit of a factor model. It compares the observed covariance matrix with a theoretically proposed covariance matrix	Chi-square test of model fit has been assessed to be overly sensitive to sample size and to vary when dealing with non-normal variables. Hence, the use of non-normal data, a small sample size (*n* = 180–300), and highly correlated items make the chi-square approximation inaccurate. An alternative to this is to use the Satorra-Bentler scaled (mean-adjusted) difference chi-squared statistic. The DIFFTEST has been recommended for models with binary and ordinal variables	(2, 93)
Root Mean Squared Error of Approximation (RMSEA)	RMSEA is a measure of the estimated discrepancy between the population and model-implied population covariance matrices per degree of freedom (139).	Browne and Cudeck recommend RMSEA ≤ 0.05 as indicative of close fit, 0.05 ≤ RMSEA ≤ 0.08 as indicative of fair fit, and values >0.10 as indicative of poor fit between the hypothesized model and the observed data. However, Hu and Bentler have suggested RMSEA ≤ 0.06 may indicate a good fit	(26, 96–100)
Tucker Lewis Index (TLI)	TLI is based on the idea of comparing the proposed factor model to a model in which no interrelationships at all are assumed among any of the items	Bentler and Bonnett suggest that models with overall fit indices of < 0.90 are generally inadequate and can be improved substantially. Hu and Bentler recommend TLI ≥ 0.95	(95–98)
Comparative Fit Index (CFI)	CFI is an incremental relative fit index that measures the relative improvement in the fit of a researcher's model over that of a baseline model	CFI ≥ 0.95 is often considered an acceptable fit	(95–98)
Standardized Root Mean Square Residual (SRMR)	SRMR is a measure of the mean absolute correlation residual, the overall difference between the observed and predicted correlations	Threshold for acceptable model fit is SRMR ≤ 0.08	(95–98)
Weighted Root Mean Square Residual (WRMR)	WRMR uses a “variance-weighted approach especially suited for models whose variables measured on different scales or have widely unequal variances” (139); it has been assessed to be most suitable in assessing models fitted to binary and ordinal data	Yu recommends a threshold of WRMR < 1.0 for assessing model fit. This index is used for confirmatory factor analysis and structural equation models with binary and ordinal variables	(101)
Standard of Reliability for scales	A reliability of 0.90 is the minimum recommended threshold that should be tolerated while a reliability of 0.95 should be the desirable standard. While the ideal has rarely been attained by most researchers, a reliability coefficient of 0.70 has often been accepted as satisfactory for most scales	Nunnally recommends a threshold of ≥0.90 for assessing internal consistency for scales	(117, 123)

#### Bifactor modeling

Bifactor modeling, also referred to as nested factor modeling, is a form of item response theory used in testing dimensionality of a scale (102, 103). This method can be used when the hypothesized factor structure from the previous model produces partially overlapping dimensions so that one could be seeing most of the items loading onto one factor and a few items loading onto a second and/or a third factor. The bifactor model allows researchers to estimate a unidimensional construct while recognizing the multidimensionality of the construct (104, 105). The bifactor model assumes each item loads onto two dimensions, i.e., items forming the construct may be associated with more than one source of true score variance (92). The first is a general latent factor that underlies all the scale items and the second, a group factor (subscale). A “bifactor model is based on the assumption that a *f*-factor solution exists for a set of *n* items with one [general]/Global (G) factor and *f* – 1 Specific (S) factors also called group factors” (92). This approach allows researchers to examine any distortion that may occur when unidimensional IRT models are fit to multidimensional data (104, 105). To determine whether to retain a construct as unidimensional or multidimensional, the factor loadings from the general factor are then compared to those from the group factors (103, 106). Where the factor loadings on the general factor are significantly larger than the group factors, a unidimensional scale is implied (103, 104). This method is assessed based on meaningful satisfactory thresholds. Alternatively, one can test for the coexistence of a general factor that underlies the construct and multiple group factors that explain the remaining variance not explained by the general factor (92). Each of these methods can be done using statistical software such as M*plus*, R, SAS, SPSS, or Stata.

#### Measurement invariance

Another method to test dimensionality is measurement invariance, also referred to as factorial invariance or measurement equivalence (107). Measurement invariance concerns the extent to which the psychometric properties of the observed indicators are transportable (generalizable) across groups or over time (108). These properties include the hypothesized factor structure, regression slopes, intercept, and residual variances. Measurement invariance is tested sequentially at five levels—configural, metric, scalar, strict (residual), and structural (107, 109). Of key significance to the test of dimensionality is configural invariance, which is concerned with whether the hypothesized factor structure is the same across groups. This assumption has to be met in order for subsequent tests to be meaningful (107, 109). For example, a hypothesized unidimensional structure, when tested across multiple countries, should be the same. This can be tested in CTT, using multigroup confirmatory factor analysis (110–112).

An alternative approach to measurement invariance in the testing of unidimensionality under item response theory is the Rasch measurement model for binary items and polytomous IRT models for categorical items. Here, emphasis is on testing the differential item functioning (DIF)—an indicator of whether “a group of respondents is scoring better than another group of respondents on an item or a test after adjusting for the overall ability scores of the respondents” (108, 113). This is analogous to the conditions underpinning measurement invariance in a multi-group CFA (108, 113).

Whether the hypothesized structure is bidimensional or multidimensional, each dimension in the structure needs to be tested again to confirm its unidimensionality. This can also be done using confirmatory factor analysis. Appropriate model fit indices and the strength of factor loadings (cf. Table 2) are the basis on which the latent structure of the items can be judged.

One commonly encountered pitfall is a lack of satisfactory global model fit in confirmatory factor analysis conducted on a new sample following a satisfactory initial factor analysis performed on a previous sample. Lack of satisfactory fit offers the opportunity to identify additional underperforming items for removal. Items with very poor loadings (≤0.3) can be considered for removal. Also, modification indices, produced by M*plus* and other structural equation modeling (SEM) programs, can help identify items that need to be modified. Sometimes a higher-order factor structure, where correlations among the original factors can be explained by one or more higher-order factors, is needed. This can also be assessed using statistical software such as M*plus*, R, SAS, SPSS, or Stata.

A good example of best practice is seen in the work of Pushpanathan et al. on the appropriateness of using a traditional confirmatory factor analysis or a bifactor model (114) in assessing whether the Parkinson's Disease Sleep Scale-Revised was better used as a unidimensional scale, a tri-dimensional scale, or a scale that has an underlying general factor and three group factors (sub-scales). They tested this using three different models—a unidimensional model (1-factor CFA); a 3-factor model (3 factor CFA) consisting of sub-scales measuring insomnia, motor symptoms and obstructive sleep apnea, and REM sleep behavior disorder; and a confirmatory bifactor model having a general factor and the same three sub-scales combined. The results of this study suggested that only the bifactor model with a general factor and the three sub-scales combined achieved satisfactory model fitness. Based on these results, the authors cautioned against the use of a unidimensional total scale scores as a cardinal indicator of sleep in Parkinson's disease, but encouraged the examination of its multidimensional subscales (114).

#### Scoring scale items

Finalized items from the tests of dimensionality can be used to create scale scores for substantive analysis including tests of reliability and validity. Scale scores can be calculated by using unweighted or weighted procedures. The unweighted approach involves summing standardized item scores or raw item scores, or computing the mean for raw item scores (115). The weighted approach in calculating scale scores can be produced via statistical software programs such as M*plus*, R, SAS, SPSS, or Stata. For instance, in using confirmatory factor analysis, structural equation models, or exploratory factor analysis, each factor produced reveals a statistically independent source of variation among a set of items (115). The contribution of each individual item to this factor is considered a weight, with the factor loading value representing the weight. The scores associated with each factor in a model then represents a composite scale score based on a weighted sum of the individual items using factor loadings (115). In general, it does not make much difference in the performance of the scale if scales are computed as unweighted items (e.g., mean or sum scores) or weighted items (e.g., factor scores).

### Step 8: tests of reliability

Reliability is the degree of consistency exhibited when a measurement is repeated under identical conditions (116). A number of standard statistics have been developed to assess reliability of a scale, including Cronbach's alpha (117), ordinal alpha (118, 119) specific to binary and ordinal scale items, test–retest reliability (coefficient of stability) (1, 2), McDonald's Omega (120), Raykov's rho (2) or Revelle's beta (121, 122), split-half estimates, Spearman-Brown formula, alternate form method (coefficient of equivalence), and inter-observer reliability (1, 2). Of these statistics, Cronbach's alpha and test–retest reliability are predominantly used to assess reliability of scales (2, 117).

#### Cronbach's alpha

Cronbach's alpha assesses the internal consistency of the scale items, i.e., the degree to which the set of items in the scale co-vary, relative to their sum score (1, 2, 117). An alpha coefficient of 0.70 has often been regarded as an acceptable threshold for reliability; however, 0.80 and 0.95 is preferred for the psychometric quality of scales (60, 117, 123). Cronbach's alpha has been the most common and seems to have received general approval; however, reliability statistics such as Raykov's rho, ordinal alpha, and Revelle's beta, which are debated to have improvements over Cronbach's alpha, are beginning to gain acceptance.

#### Test–retest reliability

An additional approach in testing reliability is the test–retest reliability. The test–retest reliability, also known as the coefficient of stability, is used to assess the degree to which the participants' performance is repeatable, i.e., how consistent their sum scores are across time (2). Researchers vary in how they assess test–retest reliability. While some prefer to use intra class correlation coefficient (124), others use the Pearson product-moment correlation (125). In both cases, the higher the correlation, the higher the test–retest reliability, with values close to zero indicating low reliability. In addition, study conditions could change values on the construct being measured over time (as in an intervention study, for example), which could lower the test-retest reliability.

The work of Johnson et al. (16) on the validation of the HIV Treatment Adherence Self-Efficacy Scale (ASES) is a good example of the test of reliability. As part of testing for reliability, the authors tested for the internal consistency reliability values for the ASES and its subscales using Raykov's rho (produces a coefficient similar to alpha but with fewer assumptions and with confidence intervals); they then tested for the temporal consistency of the ASES' factor structure. This was then followed by test–retest reliability assessment among the latent factors. The different approaches provided support for the reliability of the ASES scale.

Other approaches found to be useful and support scale reliability include split-half estimates, Spearman-Brown formula, alternate form method (coefficient of equivalence), and inter-observer reliability (1, 2).

### Step 9: tests of validity

Scale validity is the extent to which “an instrument indeed measures the latent dimension or construct it was developed to evaluate” (2). Although it is discussed at length here in Step 9, validation is an ongoing process that starts with the identification and definition of the domain of study (Step 1) and continues to its generalizability with other constructs (Step 9) (36). The validity of an instrument can be examined in numerous ways; the most common tests of validity are content validity (described in Step 2), which can be done prior to the instrument being administered to the target population, and criterion (predictive and concurrent) and construct validity (convergent, discriminant, differentiation by known groups, correlations), which occurs after survey administration.

#### Criterion validity

Criterion validity is the “degree to which there is a relationship between a given test score and performance on another measure of particular relevance, typically referred to as criterion” (1, 2). There are two forms of criterion validity: predictive (criterion) validity and concurrent (criterion) validity. Predictive validity is “the extent to which a measure predicts the answers to some other question or a result to which it ought to be related with” (31). Thus, the scale should be able to predict a behavior in the future. An example is the ability for an exclusive breastfeeding social support scale to predict exclusive breastfeeding (10). Here, the mother's willingness to exclusively breastfeed occurs after social support has been given, i.e., it should predict the behavior. Predictive validity can be estimated by examining the association between the scale scores and the criterion in question.

Concurrent criterion validity is the extent to which test scores have a stronger relationship with criterion (gold standard) measurement made at the time of test administration or shortly afterward (2). This can be estimated using Pearson product-moment correlation or latent variable modeling. The work of Greca and Stone on the psychometric evaluation of the revised version of a social anxiety scale for children (SASC-R) provides a good example for the evaluation of concurrent validity (140). In this study, the authors collected data on an earlier validated version of the SASC scale consisting of 10 items, as well as the revised version, SASC-R, which had additional 16 items making a 26-item scale. The SASC consisted of two sub scales [fear of negative evaluation (FNE), social avoidance and distress (SAD)] and the SASC-R produced three new subscales (FNE, SAD-New, and SAD-General). Using a Pearson product-moment correlation, the authors examined the inter-correlations between the common subscales for FNE, and between SAD and SAD-New. With a validity coefficient of 0.94 and 0.88, respectively, the authors found evidence of concurrent validity.

A limitation of concurrent validity is that this strategy for validity does not work with small sample sizes because of their large sampling errors. Secondly, appropriate criterion variables or “gold standards” may not be available (2). This reason may account for its omission in most validation studies.

#### Construct validity

Construct validity is the “extent to which an instrument assesses a construct of concern and is associated with evidence that measures other constructs in that domain and measures specific real-world criteria” (2). Four indicators of construct validity are relevant to scale development: convergent validity, discriminant validity, differentiation by known groups, and correlation analysis.

Convergent validity is the extent to which a construct measured in different ways yields similar results. Specifically, it is the “degree to which scores on a studied instrument are related to measures of other constructs that can be expected on theoretical grounds to be close to the one tapped into by this instrument” (2, 37, 126). This is best estimated through the multi-trait multi-method matrix (2), although in some cases researchers have used either latent variable modeling or Pearson product-moment correlation based on Fisher's Z transformation. Evidence of convergent validity of a construct can be provided by the extent to which the newly developed scale correlates highly with other variables designed to measure the same construct (2, 126). It can be invalidated by too low or weak correlations with other tests which are intended to measure the same construct.

Discriminant validity is the extent to which a measure is novel and not simply a reflection of some other construct (126). Specifically, it is the “degree to which scores on a studied instrument are differentiated from behavioral manifestations of other constructs, which on theoretical grounds can be expected not to be related to the construct underlying the instrument under investigation” (2). This is best estimated through the multi-trait multi method matrix (2). Discriminant validity is indicated by predictably low or weak correlations between the measure of interest and other measures that are supposedly not measuring the same variable or concept (126). The newly developed construct can be invalidated by too high correlations with other tests which are intended to differ in their measurements (37). This approach is critical in differentiating the newly developed construct from other rival alternatives (36).

Differentiation or comparison between known groups examines the distribution of a newly developed scale score over known binary items (126). This is premised on previous theoretical and empirical knowledge of the performance of the binary groups. An example of best practice is seen in the work of Boateng et al. on the validation of a household water insecurity scale in Kenya. In this study, we compared the mean household water insecurity scores over households with or without *E. coli* present in their drinking water. Consistent with what we knew from the extant literature, we found households with *E. coli* present in their drinking water had higher mean water insecurity scores than households that had no *E. coli* in drinking water. This suggested our scale could discriminate between particular known groups.

Although correlational analysis is frequently used by several scholars, bivariate regression analysis is preferred to correlational analysis for quantifying validity (127, 128). Regression analysis between scale scores and an indicator of the domain examined has a number of important advantages over correlational analysis. First, regression analysis quantifies the association in meaningful units, facilitating judgment of validity. Second, regression analysis avoids confounding validity with the underlying variation in the sample and therefore the results from one sample are more applicable to other samples in which the underlying variation may differ. Third, regression analysis is preferred because the regression model can be used to examine discriminant validity by adding potential alternative measures. In addition to regression analysis, alternative techniques such as analysis of standard deviations of the differences between scores and the examination of intraclass correlation coefficients (ICC) have been recommended as viable options (128).

Taken together, these methods make it possible to assess the validity of an adapted or a newly developed scale. In addition to predictive validity, existing studies in fields such as health, social, and behavioral sciences have shown that scale validity is supported if at least two of the different forms of construct validity discussed in this section have been examined. Further information about establishing validity and constructing indictors from scales can be found in Frongillo et al. (141).

## Conclusions

In sum, we have sought to give an overview of the key steps in scale development and validation (Figure 1) as well as to help the reader understand how one might approach each step (Table 1). We have also given a basic introduction to the conceptual and methodological underpinnings of each step.

Because scale development is so complicated, this should be considered a primer, i.e., a “jumping off point” for anyone interested in scale development. The technical literature and examples of rigorous scale development mentioned throughout will be important for readers to pursue. There are a number of matters not addressed here, including how to interpret scale output, the designation of cut-offs, when indices, rather than scales, are more appropriate, and principles for re-testing scales in new populations. Also, this review leans more toward the classical test theory approach to scale development; a comprehensive review on IRT modeling will be complementary. We hope this review helps to ease readers into the literature, but space precludes consideration of all these topics.

The necessity of the nine steps that we have outlined here (Table 1, Figure 1) will vary from study to study. While studies focusing on developing scales *de novo* may use all nine steps, others, e.g., those that set out to validate existing scales, may end up using only the last four steps. Resource constraints, including time, money, and participant attention and patience are very real, and must be acknowledged as additional limits to rigorous scale development. We cannot state which steps are the most important; difficult decisions about which steps to approach less rigorously can only be made by each scale developer, based on the purpose of the research, the proposed end-users of the scale, and resources available. It is our hope, however, that by outlining the general shape of the phases and steps in scale development, researchers will be able to purposively choose the steps that they will include, rather than omitting a step out of lack of knowledge.

Well-designed scales are the foundation of much of our understanding of a range of phenomena, but ensuring that we accurately quantify what we purport to measure is not a simple matter. By making scale development more approachable and transparent, we hope to facilitate the advancement of our understanding of a range of health, social, and behavioral outcomes.

## Author contributions

GB and SY developed the first draft of the scale development and validation manuscript. All authors participated in the editing and critical revision of the manuscript and approved the final version of the manuscript for publication.

### Conflict of interest statement

The authors declare that the research was conducted in the absence of any commercial or financial relationships that could be construed as a potential conflict of interest.

## Abbreviations

A-CASI: audio computer self-assisted interviewing
ASES: adherence self-efficacy scale
CAPI: computer assisted personal interviewing
CFA: confirmatory factor analysis
CASIC: computer assisted survey information collection builder
CFI: comparative fit index
CTT: classical test theory
DIF: differential item functioning
EFA: exploratory factor analysis
FIML: full information maximum likelihood
FNE: fear of negative evaluation
G: global factor
ICC: intraclass correlation coefficient
ICM: Independent cluster model
IRT: item response theory
ODK: Open Data Kit
PAPI: paper and pen/pencil interviewing
QDS: Questionnaire Development System
RMSEA: root mean square error of approximation
SAD: social avoidance and distress
SAS: statistical analysis systems
SASC-R: social anxiety scale for children revised
SEM: structural equation model
SPSS: statistical package for the social sciences
Stata: statistics and data
SRMR: standardized root mean square residual of approximation
TLI: Tucker Lewis Index
WASH: water, sanitation, and hygiene
WRMR: weighted root mean square residual.
